# Inequalities in severe COVID-19 outcomes in Wales, UK

**DOI:** 10.23889/ijpds.v9i5.2558

**Published:** 2024-09-18

**Authors:** Lucy Robinson, Matthew Curds, Ashley Akbari, Hoda Abbasizanjani, Stuart Bedston

**Affiliations:** 1 Welsh Government; 2 Swansea University

## Objective

Research evidence shows that COVID-19 has disproportionately affected people from more deprived backgrounds. In Wales (UK), there is strong evidence of a socioeconomic gradient in total hospitalisations, critical care admissions and deaths.

This study focussed on a cohort of the Welsh population using anonymised individual-level linked population-scale data in the SAIL Databank.

## Approach

This analysis examines the impact of confounding/mediating factors on severe health outcomes. We used logistic regression, considering the Welsh Index of Multiple Deprivation (WIMD) 2019 as a predictor, to investigate how the coefficient for WIMD is changed when other variables (such as comorbidities, smoking status, Body Mass Index) are added, aiming to understand whether inequalities are mediated by observed characteristics or related to other unobserved factors.

## Results

Initial findings show that age-standardised rates of hospital admissions, Intensive Care Unit (ICU) admissions and deaths were roughly twice as high in the most deprived vs least deprived WIMD quintile in Wales. We estimated Years of Life Lost per 1,000 due to COVID-19 as 28.9, with variation between WIMD quintiles of 38.4 for the most deprived and 22.2 for the least deprived. The project also produced regression formulas to evaluate the contribution of the different confounding factors.

## Conclusion

This study built on existing research findings and further explored the potential reasons for known socioeconomic inequalities in relation to COVID-19 outcomes. Insights from this study have broadened the knowledge around the impact of mediating factors and may contribute to future pandemic preparedness and the formation of future policies.

